# News and Coming Events

**Published:** 1908-08-01

**Authors:** 


### News and Coming Events.

The Prime Minister of Nepal has made a donation of £500 to the King Edward VII. Hospital Fund.

Mr. T. W. Todd, M.B., Ch.B., has been appointed Senior Demonstrator in Anatomy at Manchester University.

Mr. E. M. Corner, F.R.C.S. has been appointed surgeon to in-patients at the Great Ormond Street Children's Hospital.

Contributions received during the past week have raised the total collected on behalf of the Metropolitan Hospital Sunday Fund to about £40,000.

Messrs. Aslett Baldwin, F.R.C.S., and Gordon Watson, F.R.C.S., have been elected to the surgical staff of St. Mark's Hospital for Fistula and Diseases of the Rectum.

The president and vice-president of the Royal College of Surgeons, on July 28, sent a letter of congratulation on behalf of the council to Mr. Jonathan Hutchinson, on the occasion of his eightieth birthday.

By the will of the late Mr. Samuel Cook, of Hove, the following hospitals receive legacies of £200 each, failing the attainment of an interest by his children: The German Hospital, Dalston; the London Hospital; the Metropolitan Hospital, Kingsland Road; and the City of London Hospital, Victoria Park.

Mr. George Alexander has kindly placed the St. James's Theatre at the disposal of the Committee of the Royal National Orthopædic Hospital for a morning performance to be held in the last week of October or the first week in November, on behalf of the special appeal for £25,000 which the hospital is making to complete its new buildings in Great Portland Street.

The report of the Director General of the Army Medical Service on the progress of the Territorial Medical Service up to July 18, has been issued as a Parliamentary paper. The scheme, Sir Alfred Keogh states, has been altered, and in some respects elaborated, by consultation with the medical profession. In the course of his communications with leading members of the profession in various centres throughout the United Kingdom, Sir Alfred obtained abundant evidence to show how widely they are aware of the necessity of acquiring special knowledge of military organisation and administration.

At the Liverpool School of Tropical Medicine it has been decided to extend for the future the duration of each course of instruction from ten weeks to thirteen, a change rendered necessary by the recent advances in the subject. There will in consequence be but two courses each year, beginning on January 6 and September 13 respectively, but there will be a new short course on tropical pathology and medical entomology from June 1 to June 29 each year.

By the will of Miss Eliza Morison Hutchison, which is in course of probate, the following hospitals will benefit to the amounts stated. University College Hospital; The Brompton Hospital for Consumption; The Royal Hospital for Incurables, Putney; The Maida Vale Hospital for Epilepsy and Paralysis, £2,500 each. The London Hospital, £1,000. The Kensington Dispensary and Children's Hospital; The Great Ormond Street Children's Hospital; The Surgical Aid Society, £500 each.

We regret to announce the death, on July 28, of Sir Thomas Stevenson, M.D., senior analyst to the Home Office. Sir Thomas' name has long been familiar to the public in connection with numerous criminal cases, especially murders, and also in conection with the opening of the Druce vault last year. He edited the third edition of Taylor's "Principles and Practice of Medical Jurisprudence," and was the author also of several other works. Sir T. Stevenson was in his seventy-first year, and was knighted in 1904.

